# Stigmatization and Psychosocial Burden in Acne Patients

**DOI:** 10.1111/jocd.16678

**Published:** 2024-12-18

**Authors:** Mustafa Tunca, Zeynep Büşra Balik, Hüma Beliz Uncu, Ayşenur Botsali

**Affiliations:** ^1^ Department of Dermatology and Venereology, Gulhane Medical Faculty University of Health Sciences Ankara Turkey

**Keywords:** acne, psychodermatology, quality of life

## Abstract

**Background:**

Acne vulgaris, a common dermatological condition, has physical manifestations and significant emotional and social implications for those affected. This study aims to assess the stigma experienced by acne vulgaris patients and identify relevant physical, social, and psychological factors.

**Methods:**

The research involved individuals aged 12–45 diagnosed with acne vulgaris. The authors recorded each patient's demographic information, global acne severity, and acne scar severity. Additionally, each patient completed the Dermatologic Life Quality Index, Beck Depression Inventory, Liebowitz Social Anxiety Scale, and 6‐item stigmatization scale. Using the scale scores, the authors categorized the severity into subgroups and compared the results within these subgroups.

**Results:**

The severity of acne was positively correlated with DLQI scores (*r* = 0.196, *p* = 0.004) and stigmatization scores (*r* = 0.156, *p* = 0.024). Higher stigmatization scores were associated with increased levels of depression (*r* = 0.461, *p* < 0.001) and anxiety (*r* = 0.336, *p* < 0.001), as well as a significant impact on quality of life (*r* = 0.472, *p* < 0.001). Upon stratification of patients into three age groups (adolescence, late adolescence, and adulthood), the comparisons between these groups did not reach statistical significance.

**Conclusion:**

The results of the current study reveal a clear connection between the severity of acne and psychosocial disturbances, irrespective of age. The authors advise incorporating psychosocial screening, especially for severe acne vulgaris patients of all ages.

## 1 Introduction

Acne vulgaris (AV) is an inflammatory disease of the pilosebaceous unit, which consists of the hair follicle and sebaceous gland [1]. In addition to alterations in self‐image perception, the emotional and social implications of acne vulgaris are also profound [2]. Acne vulgaris can lead to feelings of guilt, shame, social anxiety, and social isolation [3, 4]. The psychosocial effects of acne are comparable to other dermatological conditions, such as psoriasis, which has been established for substantial quality of life impairment [5].

Studies highlight that acne patients frequently perceive themselves as less attractive and may suffer from social withdrawal and avoidance behaviors due to fear of judgment [6]. Perceived stigma among acne vulgaris patients is a critical aspect often overlooked throughout the long‐term management of AV but profoundly impacts individuals' quality of life (QoL) [7].

Perceived stigma in AV patients is predicted to increase due to recent societal norms and ideals that equate clear skin with beauty and health. Perceived stigma can extend beyond personal relationships to educational and professional settings [8]. An avoidance attitude towards social interactions or public speaking opportunities can negatively influence academic performance and career choices. A recent study showed significant appearance‐based prejudice among acne vulgaris sufferers, highlighting the reciprocal relationship between the patient and the population [9].

The quality of life impairment and mood alterations of AV patients are not directly linked to AV disease severity [10]. Some patients with acne vulgaris experience a high emotional burden despite having a very mild disease, while others with severe acne vulgaris may be at peace with their self‐image.

Addressing the perceived stigma in acne vulgaris patients requires an individualized approach that considers both physical and psychosocial determinants. The assessment should also focus on the different impacts of active inflammatory acne lesions and acne scarring. Tan et al. [11] conducted a study involving 723 adult patients with acne scars but no active acne vulgaris lesions to evaluate quality of life and dysmorphia. According to the study results, 16.9% of the participants exhibited clinical signs of dysmorphia, which was found to be associated with the severity of acne scarring.

Recognizing and addressing the psychosocial impact of acne vulgaris is vital for providing comprehensive care. This study aims to assess the perceived stigma of acne vulgaris (AV) patients and identify associated physical, social, and psychological factors using a 6‐item stigmatization scale, an objective, and specific measurement tool, and to determine the associated physical, social, and psychological factors. The relevant physical factors included the inflammatory burden related to AV and the acne scar severity. On the other hand, the psychosocial determinants were depression, anxiety, and quality of life impairment.

## 2 Materials and Methods

The study population comprised acne vulgaris patients admitted to the Dermatology Outpatient Clinic of Gülhane Training and Research Hospital, University of Health Sciences, in Ankara. All participants, or their legal guardians for those under 18, provided written informed consent. The study received ethical clearance from the local ethics committee (Approval ID: 2023/56). Exclusion criteria included pregnancy, lactation, mental retardation, prior diagnosis of psychiatric disorders, and recent use of psychoactive drugs. A single physician conducted examinations to evaluate AV disease characteristics and noted sociodemographic and clinical features such as age of onset, disease duration, marital status, previous treatments, and disease severity. Acne severity was assessed using the Global Acne Assessment Scale (GAGS), while the Goodman and Baron scar staging system was employed to determine acne scar severity [12, 13]. All patients completed the Dermatological Quality of Life Index (DLQI), Beck Depression Inventory (BDI), Liebowitz Social Anxiety Scale (LSAS), and the 6‐Item Stigmatization Scale. The validity and reliability of Turkish versions of all four tests have been successfully established [14, 15, 16, 17]. Patients were stratified into three age groups to compare the varying psychological effects of acne within these populations. These groups included adolescents (< 18 years), late adolescents (18–24 years), and adults (> 24 years).

The Dermatology Quality of Life Index is a 10‐item instrument designed to assess the psychosocial impact of dermatological conditions on patients. It evaluates skin discomfort, general health, and psychosocial well‐being over the past week. Higher scores indicate a more significant effect on the patient's quality of life [16].

The Beck depression inventory (BDI) is implemented to assess patients' depression risk or the degree of depression. Higher scores indicate a more severe level of depression. The cut‐off point determined for clinical depression is 17 [15].

The Liebowitz Social Anxiety Scale consists of 24 items used to measure fear, anxiety, and avoidance in specific social situations. Participants assess their fear/anxiety on a scale of 0 to 3 and their avoidance on a 0 to 3 scale. The total score is the sum of the scores from both subscales [17].

The 6‐item stigmatization scale measures perceived stigmatization due to skin disease using a 4‐point Likert scale. It is a component of the Impact of Chronic Skin Disease on Daily Life inventory developed by Evers et al. [14, 18].

The authors performed a categorical severity classification according to these scales' scores. For DLQI scores, there were five groups such as DLQI group 0: normal (0–1), group 1: mild [2, 3, 4, 5], group 2: moderate [6, 7, 8, 9, 10], group 3: severe [11, 12, 13, 14, 15, 16, 17, 18, 19, 20], and group 4: very severe (21–30) quality of life impairment. For BDI scores, there were six groups: group 1: normal [1, 2, 3, 4, 5, 6, 7, 8, 9, 10], group 2: mild impairment [11, 12, 13, 14, 15, 16], group 3: clinical depression [17, 18, 19, 20], group 4: moderate [21, 22, 23, 24, 25, 26, 27, 28, 29, 30], group 5: severe (31–40), and group 6: very severe depression (41–63). According to LSAS scores, there were five groups, including group 0: < 55 points, group 1: mild (55–65 p), group 2: moderate (65–80 p), group 3: severe (80–95 p), and group 4: very severe (> 95 p) anxiety and social phobia.

### 2.1 Statistical Analysis

All statistical analyses were performed using the Statistical Package for the Social Sciences (SPSS) for Windows version 22.0 (IBM, Armonk, New York). For continuous variables, results are summarized as median (minimum–maximum), and for categorical variables, frequency and percentage. Categorical variables were compared with Pearson chi‐square or Fisher's exact test. The normality of continuous variables was assessed with the Kolmogorov–Smirnov test. For continuous variables, differences between groups were determined by independent samples *t*‐test or Mann–Whitney *U* test, as appropriate. The Kruskal–Wallis *H* test was used to evaluate more than two group differences. The linear relationship between quantitative variables was performed using Spearman correlation analysis. *p* < 0.05 was considered statistically significant.

## 3 Results

The study population included 210 patients, 161 women (76.7%), and 49 (23.3%) men between 14 and 40 years old.

Thirty‐three (15.7%) patients exhibited only noninflammatory acne lesions. Global acne severity score categories were 2 in 61 (29%), 3 in 46 (21.9), 4 in 40 (19%), and 5 in 30 (14.3%) patients.

Sixty‐two (29.5%) patients were devoid of acne scars yet exhibited active acne lesions. The remaining 148 patients' Goodman Baron scar staging score categories were 1 in 82 (39%), 2 in 35 (16.7%), and 3 in 31 (14.8%). Table 1 depicts the detailed sociodemographic and clinical characteristics of the study population.

**TABLE 1:** The sociodemographic and clinical characteristics of the study population.

Variables	*n* (%)
Male/Female	49 (23.3)/161 (76.7)
Age (years) (Median (min‐max); Mean ± SD)	20 (14–40); 20.64 ± 4.42
Marital status	
Single	199 (94.8)
Married	11 (5.2)
Subgroups according to disease duration	
Less than a year	46 (21.9)
1–2 years	70 (33.3)
3–4 years	54 (25.7)
More than 5 years	40 (19.1)
Any other diagnosis of a chronic disease	
Yes	30 (14.3)
No	180 (85.7)
Any previous prescription for acne vulgaris	
Yes	165 (78.6)
No	45 (21.4)
Localization of acne lesions	
Facial	116 (55.2)
Truncal	11 (5.2)
Both facial and truncal	83 (39.5)
Acne Severity (GAGS score)	
1	33 (15.7)
2	61 (29)
3	46 (21.9)
4	40 (19)
5	30 (14.3)
Acne scar evaluation (Goodman Baron qualitative scar staging system)	
None	62 (29.5)
1	82 (39)
2	35 (16.7)
3	31 (14.8)

### 3.1 The Assessment of AV‐Specific Physical Features (GAGS and Acne Scarring)

In subgroup comparisons, acne vulgaris severity was significantly higher in patients with a disease duration of more than 5 years than in those with less than a year, 1–2 years, and 3–4 years (*p* values: 0.001, 0.001, and 0.011, respectively). The qualitative scar grading scores and global acne severity scores were directly proportional. Acne severity was significantly higher in patients with scar grade 3 (+++) than in those with scar grades 1 (+) and 2 (++) (*p* < 0.001 for both). Additionally, acne severity was significantly higher in patients with scar grade 2 (++) than in those with scar grade 1 (+) (*p* < 0.001). GAGS scores were also positively correlated with DLQI scores (*r* = 0.196, *p* = 0.004) and stigmatization scores (*r* = 0.156, *p* = 0.024). However, both of these correlations were weak [19].

The qualitative scar grading scores were higher in patients living with their family who had an established diagnosis of another disease and had previously received acne treatment (*p* values: 0.034, 0.014, and < 0.001, respectively). Lesion localization subgroups exhibited significant differences in scar formation. The qualitative scar grading scores were highest for AV patients with lesions localized to the facial region (*p* < 0.001). The patients with combined lesions within the facial and truncal skin had lower scores than patients revealing isolated facial AV lesions, and the patients with isolated truncal AV lesions had the lowest AV scores (*p* < 0.001 for each).

### 3.2 The Assessment of Psychosocial Features

The BDI scores were higher in single people than married people (*p* = 0.021). Patients with improved compliance for AV treatment exhibited lower BDI scores (*p* = 0.018). Stigmatization scores were lower in patients who had received treatment before (*p* = 0.047). No significant difference was found between the three different age groups (adolescence, late adolescence and adulthood) in terms of DLQI score, 6‐item stigmatization score, BDI score and LSAS score (*p* values, respectively, 0.219, 0.711, 0.174 and 0.618) (Table 2).

**TABLE 2:** The scores of the four scales evaluate different aspects of psychosocial impairment in different subgroups.

Variables	DLQI score	6‐item stigmatization score	BDI score	LSAS score
Total scores of the population	3 (0–22)	9 (6–24)	9 (0–43)	72 (47–182)
Gender				
Female	4 (0–22)	9 (6–24)	9 (0–41)	73 (47–182)
Male	2 (0–16)	8 (6–17)	6 (0–43)	71 (48–118)
Age (years)				
< 19	3 (0–13)	9 (6–20)	9 (0–36)	77 (48–120)
19–24	3.5 (0–22)	9 (6–20)	9.5 (0–43)	72 (47–130)
> 24	4 (0–17)	8 (6–24)	5 (0–38)	70 (48–182)
Marital status				
Single	4 (0–22)	9 (6–24)	9 (0–43)	72 (47–182)
Married	2 (0–17)	8 (7–12)	3 (0–18)	70 (48–101)
Disease duration subgroups				
Less than a year	4 (0–13)	8 (6–15)	11 (0–34)	70.5 (49–125)
1–2 years	3 (0–22)	9 (6–24)	7.5 (0–34)	69.5 (47–130)
3–4 years	4 (0–17)	10 (6–20)	8.5 (0–43)	73.5 (48–120)
More than 5 years	3 (0–17)	9 (6–17)	6 (0–41)	72 (50–182)
Any other diagnosis of a chronic disease				
Yes	2.5 (0–17)	9 (6–12)	6.5 (0–38)	73 (49–125)
No	4 (0–22)	9 (6–24)	9 (0–43)	72 (47–182)
Acne Severity (GAGS score)				
1	3 (0–11)	8 (6–14)	7 (0–31)	76 (49–118)
2	2 (0–17)	8 (6–17)	10 (0–41)	72 (48–125)
3	4.5 (0–16)	9 (6–20)	8 (0–38)	74 (48–130)
4	3 (0–22)	9.5 (6–24)	5.5 (0–43)	71.5 (47–182)
5	5 (0–16)	9 (7–20)	11 (0–36)	69 (48–120)
Acne scar evaluation (GBSS score)				
None	3 (0–16)	8 (6–17)	9.5 (0–43)	72 (48–120)
1	3 (0–17)	9 (6–24)	9 (0–34)	73 (47–125)
2	4 (0–22)	9 (6–20)	8 (0–28)	73 (53–182)
3	5 (0–16)	9 (6–20)	7 (0–36)	69 (48–120)

*Note:* The data is shown as Median (Min–Max).

Abbreviations: GAGS, Global acne grading severity score; GBSS: Goodman–Baron scar staging score.

Upon the intergroup comparison of the four scales' scores used for psychosocial assessment (DLQI, BDI, LSAS, 6‐item stigmatization scale), the results of all scales revealed a positive correlation. Table 3 presents the relevant correlation results.

**TABLE 3:** Correlation results of the DLQI, BDI, LSAS, and 6‐item stigmatization scale scores.

	Correlation data	DLQI score	BDI score	LSAS score
BDI score	*r*	0.441		
	*p*	< 0.001		
LSAS score	*r*	0.254	0.567	
	*p*	< 0.001	< 0.001	
6‐Item stigmatization score	*r*	0.472	0.461	0.336
	*p*	< 0.001	< 0.001	< 0.001

### 3.3 Intergroup Comparisons Between Categorical Severity Subgroups

#### 3.3.1 QoL Impairment (DLQI) Comparisons Among Depression (BDI) and Anxiety (LSAS) Scale Categorical Subgroups

The DLQI scores were higher in each of the BDI subgroups 2 (mild impairment), 3 (clinical depression), 4 (moderate), and 5 (severe) than in the BDI group 1 (normal) (*p* values: 0.001, < 0.001, 0.004 and 0.004, respectively). DLQI scores were higher in LSAS group 4 (very severe) than in LSAS groups 0 (normal), 1 (mild), and 2 (moderate anxiety) (*p* values: < 0.001, 0.003, and 0.001, respectively). The DLQI score was also significantly higher in LSAS group 3 (severe anxiety) than in LSAS group 0 (*p* = 0.040).

#### 3.3.2 Depression Score Comparisons (BDI) Among QoL (DLQI) and Anxiety Scale (LSAS) Categorical Subgroups

BDI scores were higher in DLQI groups 1 (mild QoL impairment), 2 (moderate QoL impairment), 3 (severe QoL impairment), and 4 (very severe QoL impairment) than in DLQI group 0 (normal QoL) (*p* values: 0.036 for group 4, < 0.001 for the others). BDI scores were higher in DLQI group number 2 (moderate QoL impairment) compared to group number 1 (mild QoL impairment) (*p* = 0.022).

BDI scores were higher in LSAS groups 1 (mild), 2 (moderate), 3 (severe), and 4 (very severe) than in LSAS 0 (normal) (*p* values: 0.016, < 0.001, < 0.001, < 0.001, respectively). Through the evaluation of LSAS group 1's (mild) BDI scores against Group 2 (moderate), group 3 (severe), and group 4 (very severe), group 1 exhibited lower BDI scores (*p* values: 0.001, < 0.001, < 0.001, respectively). The BDI scores in LSAS groups 3 (severe) and 4 (very severe) were significantly higher than those in LSAS group 2 (moderate) (*p* values: 0.029 and < 0.001, respectively). LSAS group 4's (very severe) BDI scores were also higher than group 3 (severe) (*p* = 0.007).

#### 3.3.3 Anxiety Score (LSAS) Comparisons Among QoL (DLQI) and Depression Scale (BDI) Categorical Subgroups

DLQI groups 1 (mild QoL impairment), 2 (moderate QoL impairment), and 3 (severe QoL impairment) exhibited higher LSAS scores than in group 0 (normal) (*p* values: 0.032, 0.004, 0.004, respectively). In addition, LSAS scores were higher in DLQI group 3 (severe QoL impairment) compared to group 1 (mild QoL impairment) (*p* = 0.042). LSAS scores were higher in BDI groups 2 (mild impairment), 3 (clinical depression), 4 (moderate), and 5 (severe) than in depression group 1 (normal) (*p*‐value < 0.001 for all). LSAS scores were higher in BDI group 4 (moderate depression) than in depression group 2 (mild depression) (*p* = 0.004).

#### 3.3.4 6‐Item Stigmatization Scale Score Comparisons Among QoL (DLQI), Depression (BDI), and Anxiety (LSAS) Scale (BDI) Categorical Subgroups

The stigmatization scores were higher in DLQI groups 1 (mild), 2 (moderate), and 3 (severe) than in DLQI group 0 (normal) (*p* values for all: < 0.001). Furthermore, DLQI group 3 (severe) exhibited higher stigmatization scores than DLQI group 1 (mild) (*p* = 0.004).

The stigmatization scores were higher in depression groups 2 (mild impairment), 3 (clinical depression), 4 (moderate), and 5 (severe) than in depression group 1 (normal) (*p* values: < 0.001, 0.002 < 0.001, 0.006, respectively).

The stigmatization scores of LSAS groups 3 (severe) and 4 (very severe) were higher than LSAS group 0 (normal) (p values: 0.003 and 0.001, respectively) and LSAS group 1 (mild) (p values: 0.001 and < 0.001, respectively). Additionally, LSAS group 4 (very severe) exhibited higher stigmatization scores than group 3 (severe) (*p* = 0018).

## 4 Discussion

The current study points out stigmatization as a troublesome issue in AV patients and evaluates the intricate relationship between physical factors such as AV severity and the presence of scars and psychosocial factors such as mood alterations, quality of life impairment, and also stigmatization. AV severity was positively correlated with the stigmatization scores. Patients with higher stigmatization scores also exhibited higher depression and anxiety scores with more severe quality of life impairment. These findings are consistent with the results of the research conducted by Davern and O'Donnell [20], which emphasized the relationship between perceived stigma and higher levels of psychological distress and somatic symptoms in AV patients.

The recent psychological data demonstrates that the importance of physical appearance tends to decrease with age, yet the current study's psychometric measurement results were similar in three different age groups, indicating adolescence, late adolescence, and adulthood [21]. The available literature provides conflicting results on the effect of age on the psychosocial impairment experienced by acne sufferers. While Liasides and Apergi reported that the older adults of their study population felt less unattractive and dissatisfied with their appearance due to acne, in an older study by Lasek and Chren, the results were exactly the opposite [22, 23]. The shifts in social dynamics, the increasing use of make‐up for camouflage, and also cultural differences might explain the different results of these studies conducted at different time points by including patients from various cultural backgrounds. The current study results indicate that the comparisons of psychological evaluation results did not reach statistical significance in a sample including a wide range of ages. This warrants the evaluation of psychological disturbances in all AV patients, regardless of age.

Only scarce data exist on the impact of stigmatization on AV patients. While some studies only included patients with acne scars devoid of active acne lesions, the current study evaluated acne and acne scar severity together with DLQI, BDI, LSAS, and stigmatization scales. According to the current study results, there was a positive correlation between acne severity and DLQI and stigmatization scores. The impact of AV goes beyond visible symptoms, as demonstrated by studies on acne scarring and its psychosocial effects. However, acne scar severity did not correlate with the current study's evaluation criteria. This result suggests that the psychosocial burden of AV is more pronounced throughout the active inflammatory phase than the burnout phase. However, Tan et al.'s study results indicate the independent effect of acne scarring on quality of life measures and dysmorphia [11]. The current cohort's controversial results might also be related to the fact that only a tiny proportion of the study population had moderate to severe acne scarring (31.4%).

The current study's results suggest that the severity of acne vulgaris is directly linked to quality of life impairment and stigmatization without a direct association with depression and anxiety.

On the other hand, depression, anxiety, stigmatization, and quality of life impairment measures revealed a positive correlation.

In their study, which included 100 AV cases, Hazarika and Archana reported that AV had a significant psychosocial impact [24]. Similarly, Vilar, Santos, and Sobral Filho [4] compared the QoL scores of adolescents with AV to adolescents without AV and noted apparent quality of life impairment within the AV cohort. Furthermore, the DLQI has been implemented as a dynamic tool in studies evaluating the efficacy of acne scar treatments, with significant improvement observed after therapy [25].

Acne patients, measured by LSAS, exhibit higher avoidance scores and are more negatively affected in their social, professional, and family lives [26]. Similarly, significantly higher levels of social anxiety have been reported in acne patients compared to healthy controls, as assessed by the LSAS [27]. In addition, the high prevalence of anxiety in acne patients was noted. The authors recommended incorporating simple questionnaires such as the Hospital Anxiety and Depression Scale to screen mental status [28].

The stigma experienced by AV patients is a critical problem supported by various studies. Research by Szepietowska et al. [29] has shown that AV patients do indeed encounter higher levels of stigma compared to those without acne, indicating social and psychological difficulties associated with the condition. This stigma is often fueled by misconceptions and myths about acne, not only among patients but also among healthcare providers [28]. AV patients frequently exhibit social withdrawal and social phobia, which contributes to the general stigma associated with the disease [30]. In the current study, the authors defined categorical severity groups, and the patients within the severe and very severe anxiety groups had higher stigmatization scores compared to patients with normal anxiety scores and mild anxiety groups.

The limitation of this study is the relatively heterogeneous sample composed of patients admitted to a single center. Especially the results related to acne scar severity might be altered due to the low frequency of moderate and severe acne scarring cases. This design might also hamper the evaluation of different cultural effects. Although the current study evaluated the co‐morbidities of the study population, other factors that might play a role in how acne patients are affected psychologically by the disease, such as personality traits, psychological stress, diet, regular exercise, etc., weren't included. These putative co‐factors may be the reason for the weak correlation between acne severity, quality of life, and stigmatization scores. Furthermore, the absence of a control group makes it difficult to determine whether the psychological symptoms are unique to acne or common across other dermatological or medical conditions. These aspects might be covered in future research.

The findings of this study highlight the multifaceted nature of acne vulgaris and its effects on affected individuals' physical and psychological health. A combination of social, psychological, and physiological factors influences the perceived stigma of AV patients. Addressing misconceptions and understanding the systemic and psychological effects of acne vulgaris is crucial to providing comprehensive care to affected individuals. By considering the wide range of factors contributing to acne vulgaris, healthcare providers can develop holistic treatment approaches that reduce the condition's physical symptoms and psychosocial impact. According to the current study's results, the authors recommend considering psychosocial screening, especially for severe acne vulgaris patients, regardless of their age. Additionally, promoting inclusivity and diversity in beauty standards through media representation and advocacy campaigns can challenge prevailing stereotypes and empower individuals with acne to embrace their self‐worth beyond their skin condition.

## Author Contributions

M.T., A.B. to the conception or design of the work; M.T., A.B., H.B.U., Z.B.B. to the acquisition, analysis, and interpretation of data for the work M.T., Z.B.B., A.B. drafting the work and revising the manuscript critically for important intellectual content and M.T., A.B., H.B.U. and Z.B.B. approval of the final version of the manuscript. Publication status: on behalf of all the authors that the work has not been published and is not being considered for publication elsewhere.

## Conflicts of Interest

The authors declare no conflicts of interest.

## Data Availability

The data that support the findings of this study are available from the corresponding author upon reasonable request.
